# The Type 1 Diabetes - HLA Susceptibility Interactome - Identification of HLA Genotype-Specific Disease Genes for Type 1 Diabetes

**DOI:** 10.1371/journal.pone.0009576

**Published:** 2010-03-05

**Authors:** Caroline Brorsson, Niclas Tue Hansen, Regine Bergholdt, Søren Brunak, Flemming Pociot

**Affiliations:** 1 Hagedorn Research Institute and Steno Diabetes Center, Gentofte, Denmark; 2 Center for Biological Sequence Analysis, Technical University of Denmark, Lyngby, Denmark; 3 Department of Clinical Sciences, Lund University, Malmö, Sweden; 4 Department of Biomedical Sciences, University of Copenhagen, Copenhagen, Denmark; University of Bremen, Germany

## Abstract

**Background:**

The individual contribution of genes in the HLA region to the risk of developing type 1 diabetes (T1D) is confounded by the high linkage disequilibrium (LD) in this region. Using a novel approach we have combined genetic association data with information on functional protein-protein interactions to elucidate risk independent of LD and to place the genetic association into a functional context.

**Methodology/Principal Findings:**

Genetic association data from 2300 single nucleotide polymorphisms (SNPs) in the HLA region was analysed in 2200 T1D family trios divided into six risk groups based on *HLA-DRB1* genotypes. The best SNP signal in each gene was mapped to proteins in a human protein interaction network and their significance of clustering in functional network modules was evaluated. The significant network modules identified through this approach differed between the six HLA risk groups, which could be divided into two groups based on carrying the DRB1*0301 or the DRB1*0401 allele. Proteins identified in networks specific for DRB1*0301 carriers were involved in stress response and inflammation whereas in DRB1*0401 carriers the proteins were involved in antigen processing and presentation.

**Conclusions/Significance:**

In this study we were able to hypothesise functional differences between individuals with T1D carrying specific *DRB1* alleles. The results point at candidate proteins involved in distinct cellular processes that could not only help the understanding of the pathogenesis of T1D, but also the distinction between individuals at different genetic risk for developing T1D.

## Introduction

Type 1 diabetes (T1D) is a disease of complex aetiology believed to be influenced by multiple genetic and environmental risk factors. The major genetic signal for T1D is located in the human leukocyte antigen (HLA) region on the short arm of chromosome 6 (6p21.3). The contribution of this region to the risk of developing T1D has been known for over thirty years [1], however the causative variant or variants have yet not been fully identified. The major susceptibility for T1D has been mapped to the HLA class II genes *HLA-DQB1, -DQA1* and *-DRB1* [2], [3]. Both susceptible and protective DR-DQ haplotypes exist in all populations [4]. Associations from other genes in the region have also been reported [5]–[11], however the extremely high linkage disequilibrium (LD) covering a large part of the HLA region makes it difficult to detect independent effects of individual genes [3] and these studies may have suffered from lack of statistical power to identify true effects due to small sample sizes and not dense enough genotyping. Recently novel statistical methods have been applied to genetic association data from the HLA region in T1D, and this has made it possible to identify effects of other genes independently of the effects at the classical *HLA-DR, -DQ* risk loci. These include *HLA-B* and *HLA-A*, located telomeric of the classical loci, and loci within the HLA class III region [12]–[16]. Still these new loci together with the *HLA-DQB1, -DQA1* and *-DRB1* loci cannot explain the full effect of this region. Additional loci with smaller or rarer effects are likely to exist. To detect such variants, we have focused on the development of an approach that investigates functional protein-protein interactions encoded by genes within the HLA region.

Functional relationships (e.g. co-expression, structural similarity, physical interactions) between proteins have been used to infer phenotypic effects of mutations in their corresponding genes. A thorough benchmark of the predictive power of different types of relationships showed that proteins involved in the same protein complexes had the highest tendency to also cause the same phenotype when mutated [17]. Thus, proteins interacting physically tend to be involved in similar phenotypes, which has been shown in several species [18]–[21]. With several large-scale protein-protein interaction screens across different organisms being available, protein-protein interactions can now be applied systematically to discover new disease genes [22], [23].

In this study we have used an approach that combines genetic association data with information on protein-protein interactions to identify additional genes within the HLA region that contribute to the pathogenesis of T1D. Information on protein interactions for genes that contribute to susceptibility of complex diseases may give valuable clues of not only the functional implication of candidate genes but also about additional candidates. The genetic data was generated by the Type 1 Diabetes Genetics Consortium (T1DGC), in an effort to fine map the HLA region in a large cohort of T1D families collected world-wide, but mostly of Caucasian ethnicity. We have previously validated the method in a study of a subset of the T1DGC families in order to test whether information on functional interaction pathway could help explain the effects of the HLA region on the risk of developing T1D [24]. Here we expand the study to contain over 2200 affected offspring trios and test whether different *HLA-DRB1* genotypes carried by the diabetic offspring could confer different risk due to underlying differences at a functional level.

## Methods

### Ethics Statement

All study participants, their parents or guardians gave written consent to participate and the study protocol was approved by relevant Ethics Committees and Institutional Review Boards in each country contributing family material to the study (Danish: Den Videnskabsetiske Komité for Københavns Amt # KA 04090g).

### Samples

The dataset generated by the T1DGC comprised 2321 T1D affected sib-pair families (release 2007.02.MHC). The samples comprised families from nine cohorts, Asia Pacific (AP), British Diabetes Association (BDA), Denmark (DEN), Europe (EUR), Human Biological Data Interchange (HBDI), Joslin (JOS), North America (NA), Sardinia (SAR) and United Kingdom (UK). All diabetic individuals were diagnosed before 35 years of age with continued insulin treatment since diagnosis.

### Genotyping

Genotyping was performed on two chip arrays (OPA1 and OPA2) of 1536 SNPs each from the Illumina Golden Gate technology (San Diego, CA, USA). 115 SNPs were in common on the two chips resulting in 2957 unique SNPs distributed throughout the 4 Mb classical HLA region. In addition to the SNP genotyping all individuals were also genotyped for alleles in classical HLA genes (*HLA-A, HLA-B, HLA-Cw, HLA-DPA1, HLA-DPB1, HLA-DQA1, HLA-DQB1* and *HLA-DRB1*) using PCR-based sequence-specific oligonucleotide probes and line strips from Roche Molecular Systems (Pleasanton, CA, USA). The genotyping was presented at a four-digit level except for the BDA cohort. For details on DNA samples, genotyping and QC provided by the T1DGC see [25].

### HLA-DRB1 Stratification

We used the information on *HLA-DRB1* genotypes to stratify the probands according to carriage of the *0301, *0401 or non-*0301/non-*0401 alleles (here called X). For the probands that did not have four-digit genotyping their two-digit genotype 03 or 04 was used for stratification. This divided the probands and their two parents into six different genotype groups: DR*0301/DR*0401 (DR3/DR4), DR*0401/DR*0401 (DR4/DR4), DR*0301/DR*0301 (DR3/DR3), DR*0401/DRX (DR4/DRX), DR*0301/DRX (DR3/DRX) and *DRX/DRX* ranked according to their conferred risk of T1D.

### Statistical Analyses

Individuals were removed for low genotyping success rate (<90%). SNPs were removed for low genotyping success rate (<95%), a minor allele frequency less than 1%, deviation from Hardy Weinberg equilibrium (p<0.001) in controls, and if Mendelian errors were detected. Individuals that were removed for low genotyping reduced the number of full affected offspring trios available for analysis to 2214. They were analysed for association for the remaining SNPs using the transmission disequilibrium test (TDT) [26]. All genetic analyses were performed using the program PLINK [27]. The six datasets were analysed separately for association by TDT using the quality criteria mentioned. This resulted in 589 trios being analysed for 2317 SNPs in the DR3/DR4 group (26.6%), 145 trios for 2295 SNPs in DR4/DR4 (6.5%), 202 trios for 2289 SNPs in DR3/DR3 (9.1%), 444 trios for 2369 SNPS in DR4/DRX (20.1%), 570 trios for 2358 SNPs in DR3/DRX (25.8%) and 264 trios for 2389 SNPs in the DRX/DRX group (11.9%). More than 97% of the probands in the highest risk group, DR3/DR4, carried the high risk DQB1*0302 allele associated with the DR4-DQ8 haplotype.

### Protein-Protein Interaction Networks

Protein interaction experiments are plagued by high numbers of false positive interactions and often low coverage. In order to derive a high-quality human interactome (the Inweb), we 1) combined several large scale protein-protein interaction databases, 2) inferred protein-protein interactions from model organisms, and 3) defined a quality control score for each interaction. For the purpose, we integrated interactions in 23,296 peer-reviewed articles retrieved from large protein-protein interaction databases and using the InParanoid orthology database, we inferred human protein-protein interactions from experiments in model organisms [22]. All interactions were scored and benchmarked against a gold standard to ensure that only high-confidence interactions were used in the analysis. The resulting interactome contained 313,524 unique scored protein-protein interactions covering 12,275 proteins.

### Discovering Protein-Protein Interaction Networks Involved in Diabetes

1. The resource. As the genetic analyses specifically focused on the HLA region, we derived a protein interaction network of all proteins translated from genes in this region and their respective interaction partners. Such a network is often referred to as a 2^nd^ order protein-protein interaction networks and here we will refer to the resource as the HLA-relevant protein-protein interaction network.
2. Generating protein-protein interaction modules. Each gene in the HLA-relevant protein-protein interaction network was used as bait protein in a virtual pull-down, i.e. a module consisted of a bait protein and its direct protein-protein interaction partners. This approach resulted in an inventory of modules that could be screened for association to T1D.
3. Genetic association and proteins. The TDT p-value for the SNP with the highest association to T1D was assigned to each gene including 2000 bp up- and downstream the transcription start and stop site, respectively. Thus each protein was associated to the most significant SNP lying within the gene.
4. Cleaning up modules. As the association data from the HLA region only covers ∼6% of the nodes in the HLA-relevant protein-protein interaction network, each module was reduced to only include genes with associated TDT p-values. Hereby, modules in the inventory only included gene products from the HLA region. Pull-downs with less than two associated genes were dropped.
5. Clustering of genetic association. For each protein-protein interaction module, the TDT p-values associated with each of the proteins were combined using a simple average. The combined TDT p-value for that module was then compared to a distribution of TDT p-values generated from 10,000 randomly sampled modules of the same size, i.e. the final p-value of a module was calculated as by dividing the number of random modules with a better combined p-value with 10,000.

**Figure 1:**
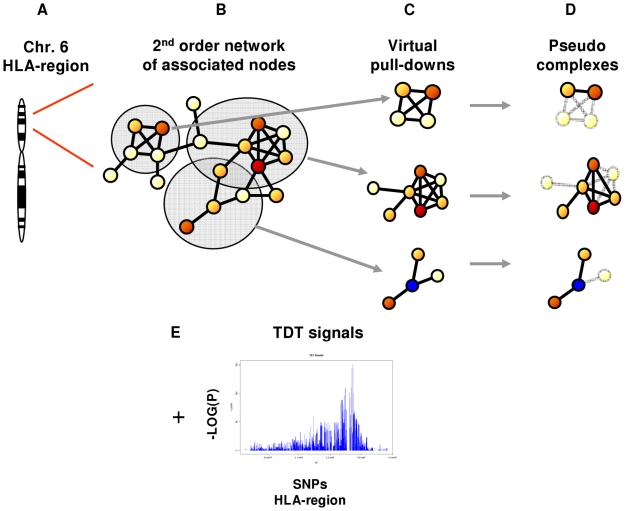
Overview of the developed approach. **A**) Genes located in the HLA region were identified and **B**) mapped to nodes or proteins in a 2^nd^ order network in the InWeb. **C**) HLA proteins and their interaction partners were used as bait to produce virtual pull-downs of network modules. **D**) Identified network modules were reduced to only contain proteins from the HLA region, as these could be associated to signals from the TDT analysis. **E**) SNPs from the TDT analysis were mapped to genes + 2000 bp up- and downstream the transcription start and stop site respectively. The best SNP signal for each gene was then mapped to the corresponding proteins in the network modules.

The steps 3) to 5) were repeated for each experiment for all six HLA genotype risk groups. The most significantly enriched modules were selected for thorough study. **Figure 1** gives an overview of the developed approach.

**Figure 2:**
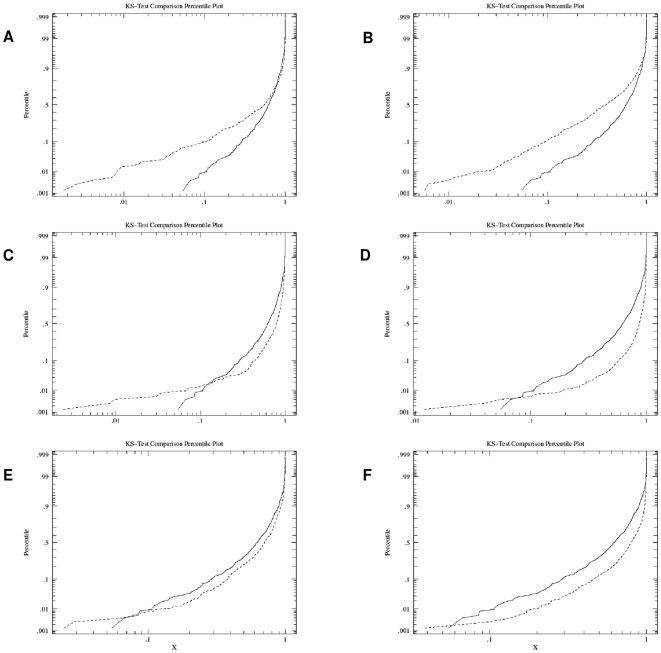
P-value distribution for HLA specific networks and the reference group. **A**) DR3/DR3: most extreme deviation higher D = 0.2083, p<0.001. **B**) DR3/DRX: most extreme deviation higher D = 0.3347, p<0.001. **C**) DR3/DR4: most extreme deviation lower D = 0.2890, p<0.001. **D**) DR4/DR4: most extreme deviation lower D = 0.4772, p<0.001. **E**) DR4/DRX: most extreme deviation lower D = 0.1586, p<0.001. **F**) DRX/DRX: most extreme deviation lower D = 0.3199, p<0.001. Dashed line: HLA risk group, solid line: reference group.

In order to test whether the genetic association clustered within the HLA risk groups, we clustered the genetic association across all HLA groups as a reference. A two-sample Kolmogorov-Smirnov test was used to compare the p-value distributions for each of the HLA risk groups with the reference distribution (**Figure 2**). **Table 1** lists the most significant network modules in each HLA risk group with information on bait proteins, the number of interaction partners within and outside the HLA region and p-values before and after correction with the reference.

**Table 1:** The top significant networks modules identified for each HLA risk group.

HLA risk group	Baita)	Chromosome	Whole pull down (#Proteins)	HLA proteins (#Proteins)	P-valueb)	Reference p-valuec)	Adjusted p-valued)
**DR3/DR3**	RCN2	15q23	66	5	0,0018	0,0545	0,0330
	TRAF6	11p12	258	6	0,0026	0,0615	0,0423
	Q5SP16	6p21	389	9	0,0041	0,0667	N.S
	ACTA1	1q42	117	5	0,0071	0,0848	N.S
	TNFRSF1A	12p13	161	8	0,0077	0,0855	N.S
**DR3/DRX**	HSPA1A	6p21	389	9	0,0057	0,0545	N.S
	CSE1L	20q13	466	7	0,0061	0,0615	N.S
	CAD	2p21	979	14	0,0098	0,0667	N.S
	TRAF6	11p12	258	6	0,0113	0,0848	N.S
	RCN2	15q23	66	5	0,0145	0,0855	N.S
**DR3/DR4**	HCRT	17q21	3	2	0,0024	0,0545	0.0440
	PSMB8	6p21	30	4	0,0085	0,0615	N.S
	CD4	12p13	100	6	0,0096	0,0667	N.S
	PSMB9	6p21	31	3	0,0302	0,0848	N.S
	TAP1	6p21	22	5	0,0318	0,0855	N.S
**DR4/DR4**	HCRT	17q21	3	2	0,012	0,0545	N.S
	CD4	12pter	100	6	0,0406	0,0615	N.S
**DR4/DRX**	HCRT	17q21	3	2	0,0242	0,0545	N.S
	HLA-DRA	6p21	9	3	0,0289	0,0615	N.S
**DRX/DRX**	HLA-DRA	6p21	9	3	0,0385	0,0545	N.S

a) Bait specifies the protein that was used to capture each network.

b) The p-value after permutation for each HLA risk group.

c) The p-value without HLA risk group stratification as a reference.

d) The p-value for each protein network after correction with the reference.

### Construction of Consensus Networks

Many proteins identified in the networks were present in more than one network and the number of interaction partners for these proteins was often large. To reduce the redundancy and identify interaction partners of special interest we used the information from all significant network modules within risk groups to construct consensus networks. For each HLA risk group, the number of times a gene was present at baseline in significant modules was counted. **Table 2** summarises the most frequent genes within each group. As the number of baseline significant networks varies between the HLA risk groups, all genes appearing more than half as many times in baseline significant modules as the most frequent gene were used to seed a consensus HLA risk group-specific network associated with T1D. The consensus networks were visualised using Cytoscape [28].

**Table 2:** Summarization of the genes occurring most frequently in the network modules for each HLA risk group.

HLA risk group	Gene	Chromosome	Times occurring in networks
**DR3/DR3**	HSPA1A	6p21	42
	HSPA1B	6p21	42
	HSPA1L	6p21	28
	TUBB	6p21	23
	C6orf48	6p21	17
	LY6G5B	6p21	14
	DDR1	6p21	11
**DR3/DRX**	HSPA1A	6p21	21
	HSPA1B	6p21	21
	TUBB	6p21	16
	LY6G5B	6p21	15
	HSPA1L	6p21	14
**DR3/DR4**	TAP2	6p21	3
	PSMB8	6p21	3
	HLA-DQA1	6p21	3
	TAP1	6p21	3
	PSMB9	6p21	2
	HLA-DQB1	6p21	2
**DR4/DR4**	HLA-DQA1	6p21	2
	HLA-DQB1	6p21	2
**DR4/DRX and DRX/DRX**	HLA-DRB1	6p21	1
	HLA-DRB5	6p21	1
	HBB	11p15.5	1
	HLA-DMA	6p21	1
	HLA-DRA	6p21	1

## Results

205 genes from the HLA region could be mapped to nodes (or proteins) in the Inweb protein interaction network. In total, 744 pull-downs were constructed containing genes within the HLA region. Network modules significantly enriched for proteins from the HLA region were identified for each of the six HLA risk groups separately after the randomisation. The highest number of significant modules (p<0.05) was found for the DR3/DR3 and the DR3/DRX group, with 44 and 26 modules respectively. Looking closer at these results we found that 16 of the modules were shared between the DR3/DR3 and DR3/DRX groups. Similarly, the groups DR3/DR4, DR4/DR4 and DR4/DRX also showed an overlap of significant network modules. These three groups however, contained substantially fewer significant modules compared to the groups that did not carry a DRB1*0401 allele (6, 2 and 2 modules respectively). One module was found significant in the low risk DRX/DRX group. **Figure 2** plots the distribution of p-values for the network modules for each of the six HLA risk groups compared to all HLA groups combined as reference. For each of the six HLA risk groups, we found more enrichment amongst the most significant clusters than we did when combining the samples across all HLA groups. The Kolmogorov-Smirnov test showed that the DR3/DR3 and the DR3/DRX groups were significantly more clustered than the combined sample (p<0.001), while the other four HLA risk groups generally were less clustered than the reference, except for the most associated networks. Of 81 significant networks identified after the randomisations three remained significant after adjusting the p-values with the reference, giving an FDR of 0.96. After correction we assume an FDR of 0.05 for the remaining three networks (**Table 1**).

### Proteins in Identified Network Modules

For the DR3/DR3 and DR3/DRX risk groups proteins from the HLA region that were identified in significant network modules were found to overlap substantially. One protein in particular, the heat shock 70 kDa protein A1 (*HSPA1A*), was found in all of the most significant modules for these two groups. The two proteins HLA-B-associated transcript 3 (*BAT3*) and c6orf48 were also found together in three of the networks. Similarly, the modules in common between the DR*0401 allele-carrying groups (DR3/DR4, DR4/DR4 and DR4/DRX) were found to contain proteins that occurred multiple times. These were represented by classical HLA class II proteins, such as HLA-DQB1, -DQA1, proteasome subunits 8 and 9 (*PSMB8, PSMB9*) and transporter 1, ATP-binding cassette sub-family B (MDR/TAP) (*TAP1*) and transporter 2, ATP-binding cassette sub-family B (MDR/TAP) (*TAP2*). The single significant network module in the heterogeneous DRX/DRX group contained the classical HLA proteins HLA-DRB1, -DRB5, -DMA and -DRA.

### Consensus Networks

Consensus networks for the six different HLA groups were constructed from the most abundant proteins in the significant modules to create a second order network. Their interaction partners outside the HLA region where included at the highest stringency level to visualise informative interaction pathways in which the HLA proteins are involved. The proteins and their functional implications in relation to immunity and T1D pathogenesis are summarised in **Table 3** for the DRB1*0301 associated consensus network and **Table 4** for the DRB1*0401 associated consensus networks.

**Table 3:** Genes in consensus network of the DR3/DR3 and DR3/DRX groups.

DR3/DR3 DR3/DRX				
Gene	Full name	Chromosome	GO terms	Description
***HSPA1A***	Heat shock 70 kDa protein A1	6p21.3	1) anti-apoptosis 2) mRNA catabolic process 3) response to unfolded protein 4) unfolded protein binding	Candidate gene in T1DM linked to the DR3 haplotype [37], [38]. Not replicated in a Japanese and American study [39], [40]. Up-regulated by cytokines in β-cells and primary islets [41], [42]. Increased T-cell response to and high levels of autoantibodies against HSP70 was detected in newly diagnosed children with T1D [43].
***HSPA1L***	Heat shock 70 kDa protein-like 1	6p21.3	1) response to unfolded protein	Also called *HSP70-HOM*. The sequence is 90% identical to *HSPA1A*. Polymorphisms have been associated with T1D [38].
***C6ORF48***	Chromosome 6 open reading frame 48	6p21.3	-	Differentially expressed in patients with symptomatic parvovirus B19 infection, which is coupled to high levels of circulating pro-inflammatory cytokines, arthritis and chronic fatigue syndrome [44].
***TUBB***	Tubulin beta	6p21.3	1) cell motion 2) natural killer cell mediated cytotoxicity 3) MHC class I protein binding	Tubulin alpha and beta are components of the microtubules with very diverse functions of the cell structure. TUBB has been identified as a key player involved in protein-interaction networks in T1D [24], [45].
***CSNK2B***	Casein kinase 2 beta subunit	6p21.3	1) signal transduction 2) identical protein binding 3) protein domain specific binding 4) protein serine/threonine kinase activity	Encodes a regulatory subunit controlling the activity of the CK2 protein. A constitutively active kinase, expressed in all human tissues and implicated in a wide variety of cellular functions [46]. Mostly studied in cancers and apoptosis where it was found to control caspase-mediated degradation of pro-apoptotic members of the Bcl-2 family [46]. Regulated by pro-inflammatory agents such as IL-1 and LPS [47]. Phosphorylates and modulates action of several transcription factors involved in inflammation, including NF-κB and STAT1 [47].
***DDR1***	Discoidin domain receptor tyrosine kinase 1	6p21.3	1) cell adhesion 2) protein binding 3) transmembrane receptor protein tyrosine kinase activity	Encodes a nonintegrin collagen receptor constitutively expressed on epithelial cells. Involved in differentiation of human monocytes into macrophages via a MAPK pathway [48]. Collagen-activation in cells over-expressing the DDR1b isoform lead to up-regulated expression and release of several cytokines and chemokines such as IL-1β, IL-8, MIP-1α and MCP-1. The up-regulation was dependent on NF-κB, indicating a role for DDR1 in the development of an inflammatory response [48].
***SLC5A1***	Solute carrier family 5 (sodium/glucose cotransporter), member 1	22q12.3	1) glucose transport 2) glucose:sodium symporter activity 3) protein binding	Glucose down-regulated the pro-inflammatory response in intestinal epithelial cells mediated by SLC5A1 activity, via a pathway leading to an alteration of NF-κB nuclear translocation [49]. SLC5A1 expression was, however, not altered by prolonged hyperglycaemia in coronary artery epithelial cells in streptozytocin-treated rats [50].
***MSR1***	Macrophage scavenger receptor 1	8p22	1) cholesterol transport 2) lipoprotein particle clearance 3) positive regulation of foam cell differentiation 4) receptor-mediated endocytosis 5) low-density lipoprotein binding 6) scavenger receptor activity	Genetic variations in *MSR1* was linked to hereditary forms of prostate cancers [51], [52] providing a link to inflammation. MSR1 was found to regulate tolerance against apoptotic cells in a mouse model of SLE, and SLE patients carried autoantibodies against MSR1 [53] indicating a role in autoimmunity.
***TSSK6***	Testis-specific serine kinase 6	19p13.11	1) protein amino acid phosphorylation 2) sperm chromatin condensation 3) ATP binding 4) magnesium ion binding 5) protein serine/threonine kinase activity	Most abundant in human testis but has been detected in all human tissues. Specifically phosphorylate histones H1, H2A, H2AX and H3 and form stable complexes with heat shock proteins (incl. HSP70) during spermiogenesis [54].
***OLR1***	Oxidised low density lipoprotein (lectin-like) receptor 1	12p13-p12	1) blood circulation 2) proteolysis	Oxidised-LDL up-regulates the expression of OLR1 on vascular endothelial cells [55]. Up-take of ox-LDL is highly injurious for epithelial cells and involved in atherosclerosis. Involved in antigen cross-priming in dendritic cells, in which exogenous antigens are processed via MHC class I pathway and initiate cytotoxic T-lymphocyte response mediated via HSP70 [56]. Expressed on macrophages where it may function as a scavenger receptor (see *MSR1*) [57].
***SAP30L***	Sin3A associated protein-like	5q33.2	-	Identified as a gene with expression up-regulated by TGF-β in T84 colon carcinoma cells but was found to be expressed in several human tissues. Protein sequence is 70% identical to SAP30 [58]. Associated with the Sin3A-HDAC co-repressor complex regulating gene expression by deacetylating histones [59]. HDAC inhibitors was shown to block the production of inflammatory mediators, such as NO and cytokines, in animal models of rheumatoid arthritis and SLE [60], [61]. In INS-1 cells, HDAC inhibitors were shown to prevent cytokine-induced β-cell apoptosis and impaired β-cell function by down-regulating NF-κB activity [62].
***ZNF44***	Zinc finger protein 44	19p13.2	1) protein binding	Identified in a screening of a placental genomic library for His/Cys motifs linking adjoining zinc fingers. Clones were then cross-hybridised to known zinc-finger-encoding cDNAs from a human T-cell cDNA library [63].
***COL11A1***	Collagen, type XI, alpha 1	1p21	1) collagen fibril organization 2) detection of mechanical stimulus involved in sensory perception of sound 3) visual perception 4) extracellular matrix binding 5) extracellular matrix structural constituent 6) protein binding, bridging	Type V and XI collagens are minor fibril-forming collagens with different tissue localisation but with closely related structural and biological properties [64]. *COL11A1* encodes the alpha 1 polypeptide that forms the heterotrimeric type XI collagen together with alpha 2 (XI) and alpha I (II) polypeptides.
***COL5A2***	Collagen, type 5, alpha 2	2q32	1) collagen fibril organization 2) eye morphogenesis 3) skin development	*COL5A2* encodes the alpha 2 polypeptide that forms the heterotrimeric type V collagen together with the alpha 1 (V) polypeptide and a second alpha 2 (V) polypeptide.

GO terms on biological process and molecular function from www.geneontology.org.

Abbreviations: IL, interleukin; LPS, lipopolysaccharide; NF-κB, nuclear factor-kappa beta; STAT1, signal transduction and activator of transcription 1; MAPK, mitogen-activated protein kinase; MIP-1α, macrophage inflammatory protein-1α; MCP-1, monocyte chemoattractant protein-1; SLE, systemic lupus erythematosus; TGF-β, transforming growth factor-β; HDAC, histone deacteylase; NO, nitric oxide; INS, insulinoma.

**Table 4:** Genes in consensus networks of the DR3/DR4, DR4/DR4 and DR4/DRX groups.

DR3/DR4 DR4/DR4 DR4/DRX				
Gene	Full name	Chromosome	GO terms	Description
Consensus network 1				
***PSMB8*** ** and ** ***PSMB9***	Proteasome subunit, beta type, 8 and −9	6p21.3	1) anaphase-promoting complex-dependent proteasomal ubiquitin-dependent protein catabolic process 2) negative regulation of ubiquitin-protein ligase activity during mitotic cell cycle 3) positive regulation of ubiquitin-protein ligase activity during mitotic cell cycle 4) protein binding	Involved in processing and assembling of endogenous peptides with MHC class I molecules, presented on the surface of antigen presenting cells where the antigens are recognised by CD8^+^ T-cells. PSMB8 and PSMB9 are subunits of the proteasome which is responsible for the degradation and cleavage of proteins and catalysis of antigen processing [29].
***TAP1*** ** and ** ***TAP2***	Transporter 1, ATP-binding cassette sub-family B (MDR/TAP) and 2	6p21.3	1) antigen processing and presentation of exogenous protein antigen via MHC class I, TAP-dependent 2) cytosol to ER transport 3) intracellular protein transport 4) protein complex assembly 5) ATP binding 6) homoaconitate hydratase activity 7) MHC class I protein binding 8) peptide antigen binding 9) peptide antigen-transporting ATPase activity 10) phosphate binding 11) protein heterodimerization activity 12) tapasin binding 13) transporter activity	Thereafter the peptides are transported from the cytosol into the endoplasmic reticulum (ER) by TAP molecules for assembly with MHC class I molecules. TAP1 and TAP2 encode subunits of the TAP heterodimer. Allelic variations within the *PSMB* and *TAP* genes have been studied previously in T1D, reviewed in [29].
***ABCB5***	ATP-binding cassette, sub-family B (MDR/TAP), member 5	17p15.3	1) transport	A member of a super-family of integral membrane proteins that participate in transport of various molecules such as ions, sugars and peptides in an ATP-dependent manner. Expressed in CD133-expressing progenitor cells of human epidermal melanocytes and was believed to be involved in cell fusion [65]. Two isoforms of ABCB5 were studied for their expression profiles in melanoma cell lines and normal tissues. Both isoforms were expressed in most types of melanomas, melanocytes and retinal epithelial cells but was not detected in other tissues, such as liver, spleen, thymus, colon or peripheral blood leukocytes, suggesting a pigment cell-specific expression [66].
Consensus network 2				
***HLA-DQB1*** ** and ** ***HLA-DQA1***	Major histocompatibillity complex, class II, DQ beta 1 and −DQ alpha 1	6p21.3	1) immune response 2) MHC class II receptor activity	The genetic risk of T1D conferred by alleles of *HLA-DQB1* and *HLA-DQA1* has been well studied [3]. The highest risk is conferred by alleles in the class II genes *HLA-DQB1*, -*DQA1* and -*DRB1.*
***HCRT***	Hypocretin (orexin) neuropeptide precursor	17q21	1) synaptic transmission	Hypocertin is associated with narcolepsy, a chronic disabling sleep disorder of unknown origin. The allele HLA-DQB1*0602 is associated with susceptibility to narcolepsy [67], but is known to cause dominant protection from T1D. The crystal structure of purified DQ*0602-hypocretin molecules was studied to elucidate the functional mechanisms for their different effects. Different characteristics of peptide-binding pockets where highlighted as important for the effects of this allele on the two diseases [68]. HLA-DQB1*0602 is also strongly associated with susceptibility to multiple sclerosis [69].

GO terms on biological process and molecular function from www.geneontology.org.

Abbreviations: ATP, adenosine triphosphate.

**Figure 3:**
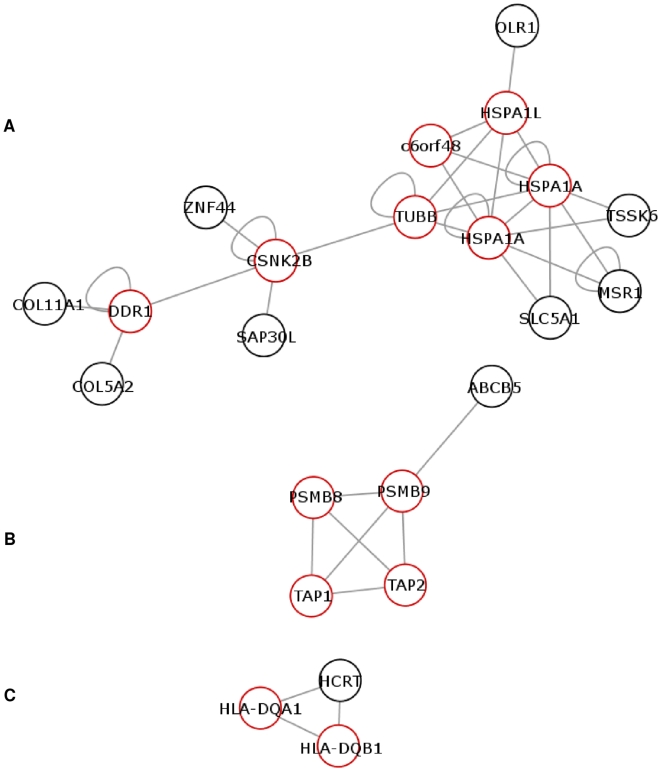
Consensus networks. **A**) displays the consensus network for the DR3/DR3 and the DR3/DRX risk groups. **B**) displays the consensus network for the DR3/DR4 and the DR4/DRX risk group. **C**) displays the consensus network for the DR4/DR4 risk group. Proteins encoded from genes in the HLA region are shown in red.

The DR3/DR3 and the DR3/DRX groups shared the same consensus network centred around six proteins from the HLA region. The HLA proteins were identified as HSPA1A, HSPA1L (old name HSP70-Hom), c6orf48, tubulin beta (*TUBB*), casein kinase 2 beta subunit (*CSNK2B*) and discoidin domain receptor tyrosine kinase 1 (*DDR1*). **Figure 3A** shows their interaction relationships within and outside the HLA region and **Table 3** summarises their information.

Two consensus networks were shared by the DR3/DR4 and the DR4/DRX group. The smallest network containing the protein hypocretin (*HCRT*) was also the consensus network for the DR4/DR4 group. Once again they contained the proteins HLA-DQB1, -DQA1, PSMB8, PSMB9, TAP1 and TAP2 and their interaction partners outside the HLA (**Figure 3B and C** and **Table 4**).

## Discussion

The complex nature of the human HLA region on chromosome 6, with the extraordinarily high LD between genes, has made it extremely difficult to elucidate the effect of individual genes for the risk of developing T1D. A recent fine mapping study of the HLA region in T1D has identified and replicated additional risk loci within the HLA class I region [12]. Independent association signals were identified for *HLA-A* and *HLA-B*, where HLA-B*039 conferred the highest risk for susceptibility. The class II-independent association of *HLA-A* and *HLA-B* has been replicated by recent studies [13]–[15]. Furthermore, evidence for independent association has been reported for the *HLA-DPB1* locus [13], [15] and for loci in the HLA class III region [16]. Other loci have failed to replicate, such as the *ITPR3* gene [10]. Most of these recent studies have used data generated by the T1DGC, and they use refined statistical methods to control for the complexity of the HLA region due to the extended LD and highly polymorphic loci. Still the divergence of some results indicates the difficulty of dissecting the independent genetic contribution of genes in this region to the risk of developing T1D. It is likely that more genes with individual but smaller or rarer effects on diabetes risk can be identified in this region. However to find these genes new approaches for analysing genetic association data are needed.

In the current study we have developed a method which combines genetic association data with information on functional protein-protein interaction networks for the corresponding genes. Using a stratification based on *HLA-DRB1* risk alleles we have identified functional protein interaction pathways that are able to discriminate between carriers of the *0301 or the *0401 risk alleles. One advantage of this approach is that it might make it possible to circumvent the issue with LD between genes as most interactions between proteins are not likely to depend on LD. However, we need to acknowledge that the extended LD between genes on chromosome 6p21 and the function of the genes within the same protein complexes make it difficult to account for LD in our analysis. Furthermore, the association signals from SNPs within genes within the HLA region are most likely confounded by the strong LD with *HLA-DRB1*, *DQA1* and *DQB1* alleles. In the developed approach we have evaluated the enrichment of T1D associated HLA proteins within interaction networks. Firstly, in the randomisation we compared the average association signal for each network to the signal from randomly generated HLA proteins networks that originated from the same TDT p-value distribution. Secondly, we compared the p-value distribution within each HLA risk group to the clustered distribution in the Kolmogorov-Smirnov test. In **Figure 2** we can see that the DR3/3 and DR3/X groups are significantly more clustered than the reference. However, also the most associated networks in each HLA risk group have lower observed p-values than expected and are thus more clustered than the reference, indicating that the networks add information to the genetic association for the HLA risk groups. The protein-protein interaction analyses performed in the current study is *in silico*, however the identified interactions are based on documented interaction data with references in the literature, weighted towards physical interactions and should be useful for guiding future biological experiments [22]. The protein-protein interaction data used in this study contained only high-confidence functional interactions, however many of the HLA proteins identified are involved in a large number of interactions with proteins outside the HLA region, as can be seen in **Table 1**. In an effort to reduce the complexity of the interactions contained in the network modules we constructed consensus networks based on the most abundant HLA proteins for each risk group and only included partners outside the HLA with a stringent criteria (see Methods).

Using this novel approach we were able to highlight functional interaction pathways for proteins that are encoded by the HLA region on chromosome 6. The common pathways that were identified for the carriers of the DRB1*0301 allele (summarised in **Table 3**) pinpoint genes and proteins involved in stress response and inflammation, such as the heat shock protein family and pathways leading to NF-κB activated gene transcription. Several of the genes that were identified as interaction partners outside the HLA region, although having diverse functions such as kinases, transcription factors and membrane transporters, are associated with roles in inflammation and thus constitute candidates with a role in the pathogenesis of autoimmunity.

In contrast the pathways identified in carriers of the DR*0401 allele (**Table 4**) highlighted proteins involved in antigen processing and transport, such as PSMB8, PSMB9, TAP1 and TAP2 as well as proteins involved in MHC class I and class II presentation. The genes *PSMB8* and *PSMB9* are located next to *TAP1* and *TAP2* in the class II region and encodes the inducible catalytic components LMP7 and LMP2 of the immunoproteasome, a specialised proteasome complex expressed upon stimulation with inflammatory cytokines. The proteasome is an enzymatic complex involved in the catalytic degradation of proteins in eukaryotic cells, including processing of antigenic peptides presented on MHC class I molecules [29]. Another important role for the proteasome is the activation of NF-κB, via processing of the NF-κB precursor and degradation of the inhibitory protein IκBα. Several studies investigating the function of the immunoproteasome in DR4-associated immune-mediated diseases have found altered functions not only in antigen processing but also in other aspects of the immune response [30]–[33].

Suggested environmental triggers of T1D have included viral infections and exposure to exogenous proteins, such as bovine insulin from cow's milk. The antigen processing pathway highlighted in the DR4-linked networks could provide indirect evidence for the involvement of exogenous proteins in the pathogenesis of immune-mediated diseases, as viral antigens are known to be processed via the immunoproteasome and presented on MHC class I molecules. Evidence for altered immunoproteasome expression and function following viral and parasite infection has also been demonstrated [34]–[36]. Taken together, these aspects of the immunoproteasome functions suggest a prominent role in regulating the immune response and pathogenesis of HLA DR-associated immune-mediated diseases.

In a previous publication [24], we proved the feasibility of using an approach that combines genetic association data with protein interaction networks using a subset of the data provided by the T1DGC. Apart from the classical *HLA* loci there is limited overlap with the network modules identified in the present study, although *CD4* was found in significant networks in both studies. The reason for this lack of overlap may be the increase in statistical power provided by the increase in trios analysed in the full dataset, as well as the stratification and focus of this study on HLA risk groups. In addition the present study focuses on the most significant network modules identified, however some of the proteins identified in [24] were found among less significant modules identified in the present study (data not shown).

Our study emphasises the importance of systems biology approaches to complement more classical statistical analyses of the genetics of T1D and other complex diseases. Genes and proteins do not exert their functions as independent entities but are part of functional protein complexes and signalling pathways. The method that we have developed gives us the opportunity to place the genetic association into a functional context, which might help us understand the underlying mechanisms and complex interplay between multiple factors contributing to disease pathogenesis. The method is not only applicable in T1D but could also be applied to association data from other common complex diseases.
